# Using communications to promote momentum toward tobacco endgame

**DOI:** 10.18332/tid/143909

**Published:** 2021-12-03

**Authors:** Megan Arendt, Shana Bedi, Isabella Costanzo, Saoimanu Sope

**Affiliations:** 1 Action on Smoking and Health, Washington, United States; 2 The Tobacco Endgame, American Heart Association, Dallas, United States; 3 Advancing Momentum for a Tobacco-Free California, Action on Smoking and Health, Washington, United States

**Keywords:** communications, endgame, social media, strategies

Tobacco endgame policies are being considered all over the world^1^, but creating a tobacco-free world will not come without strong public policy, which cannot happen without communication between tobacco control advocates, lawmakers, and the public. Communication is vital in shaping tobacco control conversations and turning the public’s attention toward a tobacco endgame. Endgame-oriented tobacco control advocacy groups such as Action on Smoking and Health (ASH) and the American Heart Association (AHA) prioritize communications work to bolster support of endgame goals.

Communications teams in tobacco control advocacy organizations should post a variety of content to promote endgame, and there are some types of content that are more effective than others. Visualizing concepts or data engages audiences more than solely relying on text^2^. For example, to illustrate phasing out the sale of commercial tobacco, consider the image of store owners tossing out or removing packs of cigarettes from their store. Imagery, unlike words or text, is excellent at showing, not telling, consumers what they need to know and can aid in showcasing the positive effects of endgame policies. When choosing voices to highlight, pull from diverse groups of people impacted by tobacco to show the depth of harm tobacco causes^3^. Prioritize true stories from real people impacted by tobacco to resonate with your audience. Professionals like doctors and scientists are helpful when looking to gain the trust of older audiences^4^. For younger audiences, feature ‘people like me’ who can share personal stories and use those experiences to support tobacco endgame policies that are most effective.

Another helpful tool for communications teams is public opinion. Citizen polls and responses to different tobacco control policies can inform how a message should be shaped to receive the best response to a message. Unfortunately, these tactics cannot ensure that a message will be positively received by everyone, and there will always be negative commenters. Policy staff must work with communications staff to ensure developed policy responses are available for genuine, thoughtful questions. When managing comments, the main goal should be to create a safe space for conversation and learning. Organizations are encouraged to develop and use an internal commenting policy to ensure all team members are monitoring social media comments in the same way to best support their brand.

Within organizations, policy staffers and communications teams should work closely together. Advocacy campaigns will not succeed without a strong communications strategy. In today’s climate especially, COVID-19 has halted in-person gatherings, making communication channels and platforms more integral. Policy staff and communications staff must be key stakeholders in all planning meetings^5^. While policy staff bring specialized information and data to the table, they need communications staff to distill that information into digestible content. Policy staff must keep their communications team informed on major policy issues and important dates and events so they can develop a plan to promote important milestones and initiatives. Policy staff can help by gathering content such as pictures and live quotes from events, data, and news for communications staff to use to promote tobacco endgame goals.

In addition to an organization’s website, social media platforms can be used to reach a broader audience. The platforms are constantly changing, so it is essential to follow trends to ensure you are active on the platform most used by your intended audience. Research the demographics of active users on each platform and take time to listen and learn best practices on that platform before building your own brand presence^6^. In general, TikTok and Instagram can be used to reach a younger audience, using creative, easy-to-edit videos to spread the organization’s message. Twitter is an excellent platform to post about breaking news and policy issues. It can also be a great forum to show your target audience that you are engaged and active by live tweeting during events. Platforms like Facebook are helpful in reaching an older audience, and it is a great place to post about news and events. Overall, there should be standardized design elements that tie all of an organization’s media together.

It is also essential for organizations to invest in endgame strategies by creating dedicated divisions and hiring staff to work on the issue. ASH and the AHA have taken these steps, and have built considerable awareness in the public health community and beyond of the possibilities for the tobacco endgame. From there, communications and policy teams across tobacco control advocacy organizations must collaborate to share knowledge, lessons learned, and amplify existing endgame policies across the US and around the world. Similar strategies were implemented during the smoke-free air movement. Civil society has strength in numbers; coalitions and advocates can gain more traction as a group than one organization on their own. Rather than competing for an audience, tobacco control advocates can work together to draw attention to tobacco endgame goals by collaborating, tagging each other, reposting other organizations’ content, and even sharing media requests to push the best messenger forward. This will help us end the tobacco epidemic faster.

Effective communication strategies are essential to the success of tobacco endgame goals. Communications teams can take the important work done by policy teams and transform it into content that continues to build up support for tobacco control. Using social media, media outreach, website resources, and communications tools can promote momentum towards the tobacco endgame.

For more information on which jurisdictions are working toward a tobacco endgame, visit https://ash.org/sunset-plans.

For more information on engaging youth in the tobacco endgame movement, visit https://www.tobaccoendgame.org/.

## Data Availability

Data sharing is not applicable to this article as no new data were created.
